# The impact of scavenging: perspective from casework in forensic anthropology

**DOI:** 10.1080/20961790.2019.1704473

**Published:** 2020-02-09

**Authors:** Douglas H. Ubelaker, Cassandra M. DeGaglia

**Affiliations:** Department of Anthropology, National Museum of Natural History, Smithsonian Institution, Washington, DC, USA

**Keywords:** Forensic sciences, forensic anthropology, scavenging, casework

## Abstract

Forensic anthropology casework frequently encounters evidence of animal scavenging associated with fragmentation and loss of skeletal material. Published research demonstrates that patterns of destruction in bone can suggest the size and type of animal involved. This study analyzes 107 cases reported on by the first author at the Smithsonian Institution to investigate patterns of scavenging in forensic anthropology casework. This investigation reveals that the extent of scavenger impact varies across the body, but primarily is concentrated in the central body area. Although extensive animal scavenging can limit analysis, some evidence of foul play can be preserved.

## Introduction

Biological anthropologists specializing in skeletal biology are uniquely trained in the skeletal anatomy of humans, which allows for analyses of remains from a wide variety of contexts. Forensic anthropologists are often asked to provide information relating to personal identification, trauma analysis, and taphonomic changes. Casework in forensic anthropology requires knowledge of skeletal biology, as well as traumatic events that leave a trace in bone. Taphonomic analysis (study of postmortem alterations) may provide key information about the postmortem interval and related postmortem events [1].

Forensic anthropologists typically study human remains following advanced decomposition and/or skeletonization. While fragmentation and separation of remains can result from human activity, they often result from scavenging with associated scattering. Recognition of non-human animal scavenging usually results from practitioners’ knowledge of the feeding behaviour of different animals common in the local region and associated patterns of destruction observed on the recovered remains [2]. Detection of scavenging and associated scattering is important to avoid confusion with human foul play, to assist in recovery, and to understand postmortem events relating to human remains. This article summarizes the recently published literature related to animal scavenging and provides perspective from the first author’s case files. This perspective offers details regarding scavenging cases, clarifying the areas of the body affected, the size of animals involved, the geographical regions of origin, association of other taphonomic variables, and evidence of trauma.

Curators at the Smithsonian Institution have a long history of casework and professional activity in forensic anthropology. As early as 1936, Aleš Hrdlička, the Smithsonian’s first curator of physical anthropology, consulted on cases at the request of the Federal Bureau of Investigation (FBI). Beginning in 1975, the first author assumed the responsibility of forensic anthropology casework originating from the FBI laboratory [3]. Smithsonian curators and others have consulted on cases originating from the FBI, regional federal and state agencies, international governments, and other organizations. Cases are brought to these biological anthropologists when specialized analysis of skeletal remains is required for which local authorities are not equipped or when expert witness testimony may be required. These cases are primarily skeletonized or severely compromised, however some include fleshy elements, radiographic images, or other components. Cases from the FBI in this sample reflect all those submitted to the organization that require anthropological analysis within the timeframe. The FBI cases originate from local, national, and international sources.

Recent research has documented considerable detail regarding the diversity of scavenger activity and the factors involved. The amount of clothing on the victim and proximity to game trails represent important variables [4] and scavenger activity varies with climate and season, as well as preference for certain species and body size [5]. Additionally, active colonization by invertebrates can inhibit feeding by vertebrates [6].

Many vertebrate species can be involved in the scavenging of human remains depending on the location of the body during decomposition. In addition to dogs and coyotes [7–9], the fox, opossum, vulture, raccoon, skunk, and crow are frequently involved in scattering and scavenging activity [6]. Omond et al. [7] report that dog scavenging of neonates features limb removal in contrast to patterns documented for larger individuals. Dog scavenging in indoor environments produces alterations to the face, neck, and arms in contrast to the pattern indicated in outdoor settings [8]. Willey and Snyder [9] report experimental results of captive timber wolves (*Canis lupus*) feeding on carcasses of deer (*Odocoileus virginianus*) in East Tennessee, US. They document a sequence of activity involving (1) movement of the carcass, opening of the thoracic cavity, and consumption of meaty sections and ribs; (2) disarticulation of one or more limbs and scattering of parts; and (3) destruction of long bone ends, the vertebral column, and rib ends. Dense bone ends and long bone diaphyses endured less damage.

Rodents frequently contribute to scavenging of human remains, as noted by Haglund [10]. The porcupine, gerbil, mouse, squirrel, and rat are all known to transport and gnaw on bones leaving behind characteristic alterations. Klippel and Synstelien [11] have called attention specifically to bone gnawing activity of the brown rat and gray squirrel. Pokines [12] documents a vole nesting site within human remains and notes that the woodland vole produces gnaw marks in bone that are consistent with their incisor size.

Raccoons represent frequent scavengers in geographic areas where they are abundant. Jeong et al. [13] report that soft tissue is usually targeted by raccoons, especially from the arms and legs. The opossum also primarily concentrates on soft tissue but can relocate small bones short distances and can produce gnaw marks and punctures in small bones and on the margins of flat bones [14].

In geographic regions where they live, vultures represent common scavengers of human remains. Their consumption of soft tissue can lead to rapid skeletonization. Reeves [15] notes that vultures can produce scratches on bone and often leave behind diagnostic feathers and faeces. However, Spradley et al. [16] indicate that scavenging can be delayed and involve considerable dispersal of elements. Reporting from southern Illinois, Dabbs and Martin [17] found variation in both the timing and extent of vulture feeding and detected no alterations of bone.

Although uncommon, cases involving shark scavenging have been reported. Shark activity is suggested by context, the pattern of injury, and recovery of fragments of shark teeth from the remains [18]. A recent review of multiple cases involving apparent shark scavenging reports patterns of “incised gouges in cortical bone”, punctures, blunt force fractures, scratches, and fine incisions [19].

Pigs are notorious scavengers but are rarely involved with human remains. However, Berryman [20] reports a case study involving pig scavenging of a human decedent. He noted characteristic tooth marks and concentration on the viscera, throat, and facial regions. Skeletal indications of pig scavenging include curvilinear scoring that differs from what is seen in carnivores or rodents due to differences in dental morphology, areas of missing cortical bone, and fragments of cortical bone displaced into the trabeculae [20]. Such cases can be challenging for forensic anthropologists.

Bears are also infrequent scavengers of human remains. When bears are involved in scavenging activities, Carson et al. [21] report that they create greater transport and removal of remains and scavenge for more prolonged periods than what is documented for smaller vertebrates. Rippley et al. [22] document a bobcat feeding on human remains producing soft tissue alterations. Meckel et al. [23] provided photographic evidence of a white-tailed deer gnawing on human bone, producing superficial grooves in cortical bone.

Dibner et al. [24] report on an experiment to characterize the scavenger community on the Hawaiian island Oahu. The authors observed that the Indian mongoose (*Herpestes javanicus*), a species not native to the Hawaiian islands, was the sole vertebrate scavenger of the carcasses. Initially, the mongooses appeared to have been attracted to the larvae colonizing the remains before transitioning to scavenging the decomposing flesh. Also described in this paper are the species of bacteria active on the remains, which provides a more holistic view of a decomposer community [24].

This study aims to provide information about scavenging patterns in forensic anthropology casework, primarily conducted in the US. While the bulk of data collected for this study is from cases originating in North America, there is research being conducted regarding patterns of scavengers from ecosystems around the world [25–30].

## Materials and methods

For this study, cases spanning 44 years from 1975–2019 were examined. This timeframe reflects cases available that were reported on by the first author. These include cases originating from the FBI as well as other agencies. Out of 987 total cases, 714 included reports relevant to taphonomic studies. Of these, 107 (15%) contained information on observed evidence of scavenging by non-human animals on examined skeletal elements. One case with indications of scavenging only on soft tissue was excluded from this study. The 107 cases with evidence of scavenging comprise the primary dataset for this study.

Evidence of scavenging was classified as having been caused by either small (weighing one pound or less) or large (weighing more than one pound) animals. The size and type (rodent tooth striations, canine pitting, etc.) of damage was used to determine the size of scavenging animals. When scavenger size was stated in original reports, that information was recorded. When scavenging was noted but the size of the scavenger or scavengers was not stated, size was discerned through examination of photographs pertaining to the case. When size was not noted and photographs were unavailable, the size designation of “indeterminate” was assigned.

In addition to the size of the scavengers, information about the area, or areas, of the body that were affected was recorded. For this study, the following body regions were designated: (1) Head (cranium and mandible), (2) Thorax (C-1 through T-12, hyoid, clavicles, scapulae, and ribs), (3) Upper extremities (humeri, ulnae, and radii), (4) Hands (carpals, metacarpals, and hand phalanges), (5) Abdomen/pelvis (L-1 through coccyx and os coxae), (6) Lower extremities (femora, patellae, tibiae, and fibulae), and (7) Feet (tarsals, metatarsals, and foot phalanges). Paired skeletal elements were further assigned to their side (e.g. right arm, left foot). When remains of paired regions were too fragmentary to allow side determination, the category of “indeterminate” was assigned. Marks of scavenging were also recorded on recovered non-human skeletal remains, using the designation of “N-H”. Animal size was examined in relation to area of body impacted to explore if different sized animals selectively scavenged particular areas of the body.

The geographic region from which the case originated also was noted. Geographic designations from the U.S. Census Bureau [31] were used to divide the US into four regions: the Northeast, the Midwest, the South, and the West. Population size of each region was not noted as the length of time covered in this study encompassed changes in population numbers. Similarly, population density was not included for the designated regions because they are large and highly variable in the density of humans living in specific areas.

Additional taphonomic factors were also examined. These included evidence of surface exposure, sun exposure, burial, exposure to water, mummification, adipocere formation, and thermal effects. Further, perimortem projectile trauma, sharp force trauma, and blunt force trauma are also included as evidence of foul play. These factors were selected due to their potential to elucidate patterns of scavenging.

Because many of the case files examined for this study did not contain information regarding the position of recovered elements in relation to each other, scattering patterns were not investigated. Statistical analysis of scavenging activity compared to environments from which remains were recovered was not conducted because information about recovery site was not consistently available for cases comprising the examined sample.

## Results

**Figure 1.**
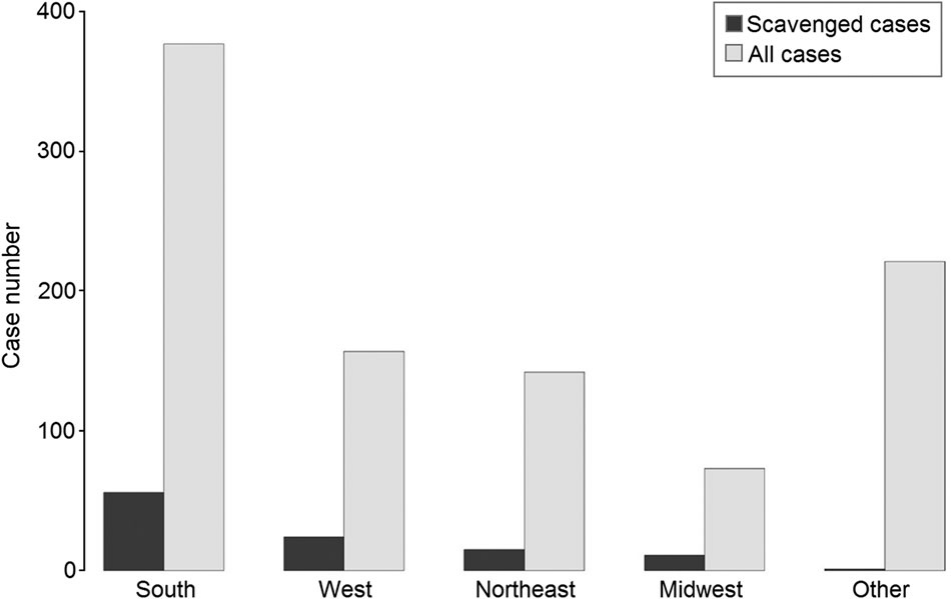
Relationship of cases with evidence of scavenging to all cases by region.

Of the 107 cases included in this study, 56 (52%) originated from the South, 24 (23%) originated from the West, 15 (14%) originated from the Northeast, and 11 (10%) originated from the Midwest. One case (1%) originated from outside of the US (Caribbean). The relationship between cases from each region and the total number of cases from that region is shown in Figure 1.

**Figure 2.**
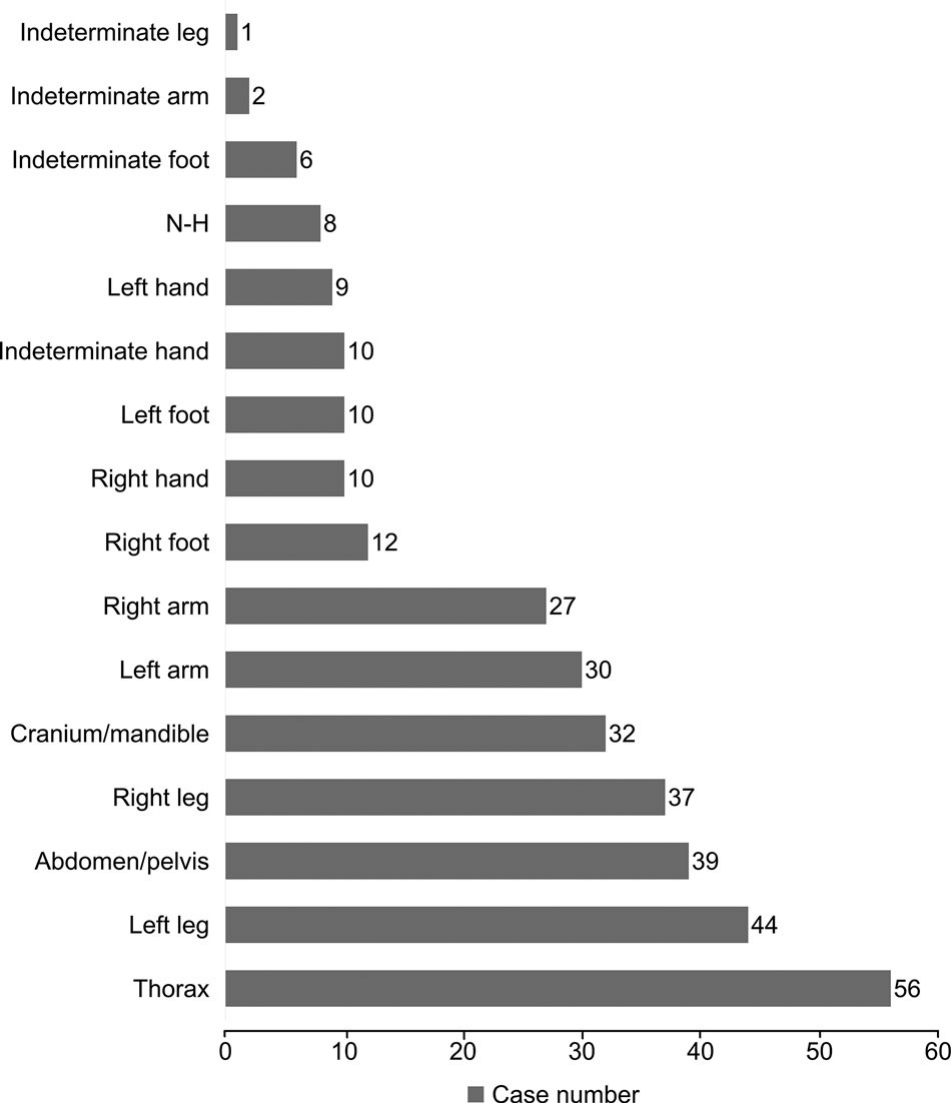
Cases with evidence of scavenging by body region. N-H: non-human.

Many cases presented evidence of scavenging on more than one anatomical region. Across the 107 cases 312 total anatomical regions displayed indications of scavenging (including the designation of “non-human”). The most commonly scavenged anatomical region was the thorax (56 cases, 52%), followed by the left leg (44 cases, 41%) and the abdomen/pelvis (39 cases, 36%). Figure 2 shows the frequency of scavenging by anatomical region, including non-human remains. It is important to note that these numbers are influenced by the amount of evidence recovered and the nature of the case, which affect the evidence submitted for analysis.

In this sample, scavenging by large animals was more prevalent than scavenging by small animals. Seventy-four cases (69%) displayed evidence of scavenging by large animals while 30 cases (28%) were affected by small scavengers and 14 cases (13%) presented evidence of scavenging by animals of an indeterminate size. Twelve cases (11%) were affected by multiple scavengers of varying sizes.

From the 107 cases, a total of 312 anatomical regions displayed evidence of scavenging by non-human animals. Scavenging by large animals was four times more common than scavenging by small animals, with 265 (85%) analyzed anatomical regions displaying evidence of scavenging by large animals and 58 (19%) displaying evidence of gnawing by smaller animals. Animals of an indeterminate size scavenged 28 (9%) of the examined anatomical regions. The total number of anatomical regions scavenged by large, small, and indeterminate animals is greater than 312 because in some cases multiple animals of different sizes scavenged the same body areas. Sixty-three cases (59%) presented evidence of scavenging on more than one anatomical region. It should be noted that this number is likely affected by the amount of evidence submitted for analysis (e.g. the authors have no record of post-cranial data on cases of skulls submitted for consultation on facial approximation).

**Figure 3.**
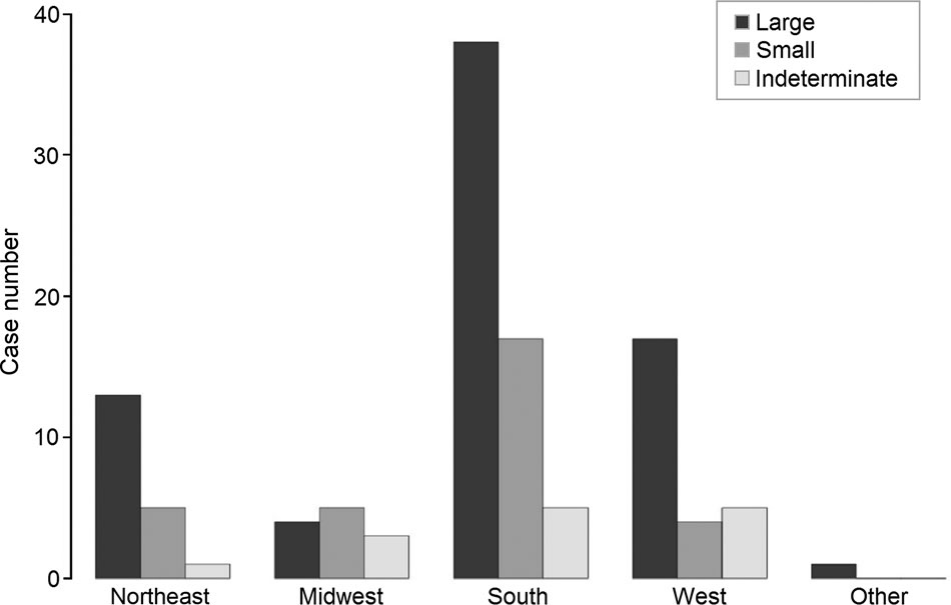
Frequency of scavenger size by geographic region.

In the Northeast, 13 cases showed evidence of large animal scavenging, five displayed trademarks of smaller animal scavenging, and one was scavenged by an animal of indeterminate size. Thirty-eight cases from the South presented evidence of scavenging by large animals while 17 showed smaller animal marks and five were indeterminate. Seventeen cases from the West were scavenged by large animals, four by smaller animals, and five by animals of an indeterminate size. In the Midwest, small scavengers were marginally more common with five cases being affected while four were altered by large scavengers and three by scavengers of an indeterminate size. The one case originating from outside of the US was affected by a large scavenger or scavengers. The total number of cases exceeds 107 due to some cases being scavenged by multiple animals of varying sizes. Figure 3 presents data on the frequency of scavenger size by geographic region.

The most common alternative taphonomic factor noted in the files associated with scavenging was evidence of surface exposure (80 cases, 75%). Sun bleaching was noted on bones from 24 cases (22%). Nine cases affected by scavenging also showed evidence of at least partial burial (8%). Nine cases (8%) from the sample included indications of water exposure. Three cases (3%) displayed adipocere formation and an additional three (3%) showed hallmarks of exposure to extremely high temperatures. One scavenged case (1%) presented a level of mummification of associated soft tissue.

Projectile and sharp force were the most common perimortem traumatic factors in the sample with 11 cases (10%) displaying projectile trauma and 11 (10%) displaying hallmarks of sharp force trauma. Three cases (3%) displayed evidence of blunt force trauma on the bones. The percentages of additional taphonomic and traumatic factors affecting cases totals more than 100% because multiple cases were affected by more than one taphonomic and/or traumatic event.

## Discussion

The regional distribution of included cases largely reflects the historical availability of forensic anthropologists. Although some cases were local to the Washington D.C. area, most originated elsewhere. Casework consultation with the first author occurred when expertise was not available locally or specialized procedures were required. The high proportion of scavenging cases originating from the South correlates with the southern origin of many overall cases (Figure 1). The historical high percentage of southern cases relates to the demand for FBI assistance from that region. That demand largely indicates the lack of consultation with local forensic anthropologists in the southern region of the US, especially during the early decades of the case history reported here.

The distribution of evidence of scavenging across body regions largely supports what is suggested in the published literature [4, 9–11]. The central body, where viscera are concentrated, is the primary scavenging target. This may indicate that scavenging activity of carnivores is more likely to occur earlier in the decomposition process when the soft tissue and viscera are fresher. Hands and feet were less frequently affected. It should be noted that this may also reflect the difficulty of recovering the small and fragile bones of these anatomical areas, particularly when scavenging activity may have relocated them a sizable distance from the rest of the remains.

Assessment of animal size suggests that most evidence of scavenging relates to large animals. However, 9% of cases presented evidence of scavenging by both small and large animals. Again, these results are consistent with the published literature.

Cases from all geographic regions except the Midwest were more frequently scavenged by large animals.

Animal scavenging requires access to human remains. Accordingly, casework analysis reveals a high incidence of evidence of scavenging with indications of surface deposit of remains. Such indications include sun bleaching and recovery information. Access was also evident from some cases involving partial burial and those recovered from aquatic environments. Given the limited amount of information regarding recovery available in the case files, additional detail is limited regarding the circumstances under which scavenging occurs.

Of the 987 total cases, 126 (13%) presented evidence of perimortem trauma, of which 23 (18%) were also scavenged, comprising 21% of the 107 cases with evidence of scavenging. These data suggest only a weak association of perimortem trauma and evidence of scavenging. However, only evidence of trauma left on bone was recorded and the possibility of traumatic events which affected only soft tissues may have encouraged scavenger activity on other body regions.

Of the 312 anatomical regions that were scavenged, 77 (25%) were from cases that presented evidence of perimortem trauma. Indications of scavenging and perimortem trauma aligned on 34 anatomical regions (11% of the 312 scavenged anatomical regions and 44% of the 77 anatomical regions from cases displaying both scavenging and perimortem trauma). Again, these data on affected anatomical regions reveal only a weak association (fewer than half) of perimortem trauma and scavenging.

Projectile, sharp force, and blunt force trauma were all represented in the scavenged sample. Blast trauma generated by explosions was a factor in several cases, however none displayed marks of scavenging, likely due to rapid recovery of the remains from the blast sites.

Although animal scavenging can lead to fragmentation and loss of evidence, indications of foul play are frequently preserved. Of the 107 cases with evidence of scavenging, 20 (19%) retained evidence of foul play.

## Conclusion

Forensic anthropology casework frequently reveals evidence of animal scavenging. The prevalence of such evidence varies regionally, reflecting climatic factors and animal presence. Scavenger activity concentrates on central body areas rather than the hands and feet. Presumably, the central body area with its abdominal viscera offers a more attractive food source. Extensive animal scavenging can limit analysis but evidence of foul play can be preserved. Perimortem trauma does not always lead to scavenging but can coincide, likely reflecting the local availability of scavenging animals.

Further research might address patterns of scavenging in more specific geographic regions, such as within a state or province. Other studies might examine possible variability in scavenger behaviours in urban and rural environments. Investigating if the thoracic and abdominal cavities are targeted earlier in the decomposition process when the viscera are fresher may prove to be important. Similarly, testing for the timing and body area preference of scavenging by smaller vertebrates, such as rats and squirrels, may provide more information to assist time since death estimation. Studies of soft tissue involvement may be useful for assessing total body area affected by scavenging activity and other research questions that are not answerable with skeletal analysis alone.
